# Butyrate reverses ferroptosis resistance in colorectal cancer by inducing c-Fos-dependent xCT suppression

**DOI:** 10.1016/j.redox.2023.102822

**Published:** 2023-07-20

**Authors:** Ying He, Yuhang Ling, Zhiyong Zhang, Randall Tyler Mertens, Qian Cao, Xutao Xu, Ke Guo, Qian Shi, Xilin Zhang, Lixia Huo, Kan Wang, Huihui Guo, Weiyun Shen, Manlu Shen, Wenming Feng, Peng Xiao

**Affiliations:** a Central Laboratory, The First Affiliated Hospital of Huzhou University, Huzhou, 313000, China; b Huzhou Key Laboratory of Translational Medicine, The First People's Hospital of Huzhou, Huzhou, 313000, China; c Department of Colorectal Surgery, The First Affiliated Hospital of Zhengzhou University, Zhengzhou, 450000, China; d Department of Immunology, Harvard Medical School, Boston, 02120, USA; e Department of Gastroenterology, Sir Run Run Shaw Hospital, Zhejiang University School of Medicine, Hangzhou, 310016, China; f Inflammatory Bowel Disease Center, Sir Run Run Shaw Hospital, Zhejiang University School of Medicine, Hangzhou, 310016, China; g Institute of Immunology, Zhejiang University School of Medicine, 310058, Hangzhou, China; h The Key Laboratory for Immunity and Inflammatory Diseases of Zhejiang Province, Hangzhou, 310058, China

**Keywords:** Colorectal cancer, Butyrate, Ferroptosis, xCT, c-Fos

## Abstract

Ferroptosis has emerged to be a promising approach in cancer therapies; however, colorectal cancer (CRC) is relatively insensitive to ferroptosis. Exactly how the gut microenvironment impacts the ferroptotic sensitivity of CRC remains unknown. Herein, by performing metabolomics, we discovered that butyrate concentrations were significantly decreased in CRC patients. Butyrate supplementation sensitized CRC mice to ferroptosis induction, showing great *in vivo* translatability. Particularly, butyrate treatment reduced ferroptotic resistance of cancer stem cells. Mechanistically, butyrate inhibited xCT expression and xCT-dependent glutathione synthesis. Moreover, we identified c-Fos as a novel xCT suppressor, and further elucidated that butyrate induced c-Fos expression via disrupting class I HDAC activity. In CRC patients, butyrate negatively correlated with tumor xCT expression and positively correlated with c-Fos expression. Finally, butyrate was found to boost the pro-ferroptotic function of oxaliplatin (OXA). Immunohistochemistry data showed that OXA non-responders exhibited higher xCT expression compared to OXA responders. Hence, butyrate supplementation is a promising approach to break the ferroptosis resistance in CRC.

## Highlights

• Butyrate increases ferroptosis sensitivity in colorectal cancer by suppressing xCT expression.
• C-Fos protein was identified as a novel xCT repressor.
• Butyrate alters the chromatin accessibility at *FOS* promoter by inhibiting class I HDAC activity.
• Butyrate breaks the ferroptosis resistance of cancer stem cells.
• Butyrate potentiates the therapeutic efficacy of oxaliplatin.

## Graphical abstract

**Figure:**
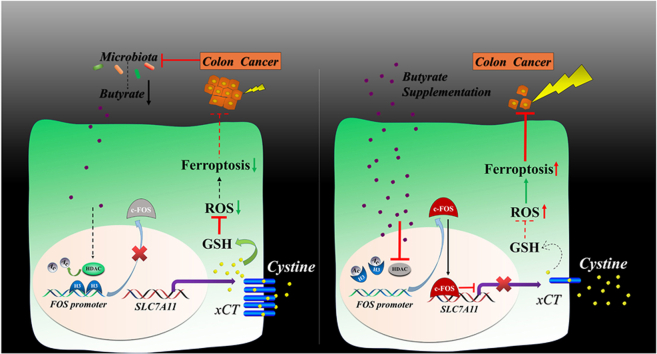

## 1 Introduction

Ferroptosis, a new form of programmed cell death which was first described in 2012 [1], is characterized by iron-dependent lipid peroxidation. The occurrence of ferroptosis is caspase-independent and could not be reversed by apoptosis and necrosis inhibitors. It is reported that malignant cells are more susceptible to ferroptosis than normal cells due to the relatively high intracellular iron levels [2]. It should be considered, however, that not all cancers are created equal. Different cancer cells can evolve multiple defensive mechanisms in order to antagonize ferroptotic cell stress depending on their type, location, and tumor microenvironment. For example, oncogenic activation of PI3K-AKT signaling desensitized tumor cells to ferroptosis. Moreover, the inactivation of p53 also led to reduced tumor ferroptosis [3]. Among various human cancers, colorectal cancer (CRC) has been shown to be relatively insensitive to ferroptosis induction, yet exactly how the colon microenvironment affects ferroptosis sensitivity remains to be elucidated.

The mammalian colon is inhabited by more than 10^13^ commensal bacteria and diverse types of bacteria-derived products. Among these, short-chain fatty acids (SCFAs) represent the most abundant metabolites generated by the fermentation of certain species of bacteria. SCFAs maintain gut homeostasis by suppressing inflammation and preserving epithelial integrity [4]. Recently, emerging roles of SCFAs in cancer therapy have been reported; however, these reports have produced controversial conclusions. For example, SCFAs inhibited the effect of anti-CTLA-4 immunotherapy [5], yet boosted the function of anti-tumor chimeric antigen receptor (CAR) T cells [6]. In addition, SCFAs were reported to either limit or increase the therapeutic efficacy of chemotherapy or radiotherapy respectively [7,8]. Therefore, the functions of SFCAs in varying types of cancer therapy necessitate further clarification.

Although microbial metabolites like SCFAs are considered to be the most prominent feature of the gut microenvironment, how they affect the outcome of ferroptosis induction is still poorly understood to this date. Herein, through performing multi-omics approaches, we provide the first evidence that the reduced concentration of butyrate in CRC patients is a crucial regulator of failure in ferroptosis induction. In addition, the underlying molecular mechanisms through which butyrate enhances CRC ferroptosis were explored in depth and validated *in vivo*.

## 2 Material and methods

### 2.1 Reagents

SCFAs were obtained from Sigma-Aldrich (St. Louis, MO, USA). Erastin, ferrostatin-1, FIN56, necrostatin-1, oxaliplatin, RSL3,pertussis toxin, Entinostat, Ricolinostat, Nicotinamide, SIS17, T5224 and TMP269 were purchased from MedChemExpress (Shanghai, China). DCFH-DA, Hoechst33342, *N*-acetyl-l-cysteine, propidium iodide, trichostatin A, and Z-vad-FMK were obtained from Beyotime (Shanghai, China). Other cytokines/chemicals included B27 (Invitrogen, Carlsbad, CA, USA), recombinant human EGF and FGF (PeproTech, Rocky Hill, NJ, USA), Cystine (Solarbio, Beijing, China), C11-BODIPY 581/591 dye (Abclonal, Wuhan, China).

### 2.2 Human specimens

Tumor samples and stool samples were collected from CRC patients in the First People's Hospital of Huzhou and Sir Run Run Shaw Hospital of Zhejiang University. The resected tumor tissues, as well as the stool samples were preserved at −80 °C after quick freezing using liquid nitrogen. For tissue array analysis, sixty-five paired paraffin-embedded colorectal carcinoma and adjacent normal tissues were collected respectively, and constructed into a tissue array block. For oxaliplatin (OXA) responsiveness analysis, paraffin-embedded primary colorectal tumors of CRC patients who presented relapse during the period of OXA treatment after surgery were collected. Experiments involving human specimens were conducted under the approval from the Medical Ethics Committee of Sir Run Run Shaw Hospital of Zhejiang University (20220103–56), and the First Affiliated Hospital of Huzhou University (2021KYLL-Y-005).

### 2.3 Cell culture and plasmid transfection

Human colon carcinoma cell lines (HCT116, SW480, SW620, and RKO) and human liver carcinoma cell line Hep3b were obtained from the Cell Bank of Chinese Academy of Sciences in Shanghai. Cell lines were authenticated using Short Tandem Repeat (STR) analysis, and were cultured in Dulbecco's Modified Eagle's Medium (SW480, SW620, RKO, Hep3b) or McCoy's 5A medium (HCT116) with 10% FBS. Stable xCT^OE^ HCT116 cells transfected with pcDNA3.4 plasmid were generated after G418 selection (400 μg/mL) and expansion of transfected cells for 2 weeks. Stable c-FOS^KD^, c-FOS^KD^xCT^KD^ or control HCT116 cells were generated by shRNA lenti-virus (pLVX-puro vector) infection (MOI 10) and then puromycin selection (0.6 μg/mL) for another 2 weeks. The interfering lenti-viruses targeted following oligonucleotides:

c-FOS^KD^, GCAATAGTGTGTTCTGATTAG;
xCT^KD^, GGTTGCCCTTTCCCTCTATTC.

To generate cancer stem-like cell spheres, HCT116 cells were seeded into ultra-low attachment 96-well plates (Corning) and cultured in serum-free McCoy's 5A medium supplemented with B27 (1:50), EGF (20 ng/mL) and FGF (10 ng/mL) as previously described [9].

For transient transfection, pcDNA3.4-c-FOS^FL^, c-FOS^DBD^, c-FOS^△DBD^, pcDNA3.4-xCT, or empty vectors were transfected into cells with Ez-Trans reagent (Life iLAB, Shanghai, China) following the manufacturer's procedure.

### 2.4 Animal models

For xenograft model, HCT116 (3 × 10^6^), SW480 (2 × 10^6^), and Hep3b (4 × 10^6^) cells were subcutaneously injected into the right flank of 6-to-8-week BALB/c nude mice, respectively. When the tumors were palpable, mice were treated with erastin (30 mg/kg weight, i.p., every three or four days), butyrate (100 mM in drinking water), or butyrate plus erastin. In another experiment, oxaliplatin (30 mg/kg weight, i.p., every three or four days) was used for mouse treatment. Tumor diameters were measured twice weekly and tumor volumes were calculated as: length × width ^2^ × 1/2.

For azoxymethane/dextran sodium sulfate (AOM/DSS) model, 6-to-8-week wild-type C57BL/6 mice were intraperitoneally injected with AOM (10 mg/kg weight, Sigma-Aldrich) once, followed by three cycles of 2.3% DSS (MP Biochemicals; 36,000–50,000 mw) administration in drinking water for 5 days. In each cycle, mice had access to normal water for two weeks after DSS administration. 80 days later, mice were treated with erastin (30 mg/kg weight, i.p., every three or four days), butyrate (100 mM in drinking water), or butyrate plus erastin. After three-week treatment, mice were sacrificed and the tumors were counted. Mice were maintained under specific-pathogen free conditions, and were randomly allocated into experimental groups. Animal experiments were performed according to protocols approved by the Institutional Animal Care and Use Committee of Zhejiang University, and the Animal Care and Use Committee of First Affiliated Hospital of Huzhou University.

### 2.5 Dual-luciferase reporter assay

HCT116 cells cultured in 24-well plates were transfected with pcDNA3.4-c-FOS or pcDNA3.4, pGL3-basic-xCT promoter or pGL3-basic, and pRL-TK using Ez-Trans reagent. Cells were lysed 48 h after transfection, the firefly and renilla luciferase activities were determined using Dual-Glo Luciferase Assay System (Promega). Results were expressed as ratio of firefly to Renilla luciferase luminescence.

### 2.6 Transmission electron microscopy

Cells in 6-well plates were fixed with 2.5% glutaraldehyde solution for 30 min and then collected using cell scrapers. Cells were fixed at 4 °C for another 24 h. Samples were sent to Servicebio Company (Wuhan, China) for resin embedding and section preparation (70 nm). Then the mitochondrial morphology was imaged using H7650 electron microscope (HITACHI, Tokyo, Japan) at 80 kV.

### 2.7 Cell viability assay

Cell viability was tested using CCK8 reagent (Dojindo, Japan) according to the manufacturer's protocol. Cells were seeded into E-plate 16 (ACEA Biosciences, USA) after baseline normalization, and then cell index was automatically collected every 30 min. During the period of drug treatment, cell index was monitored dynamically by the xCELLigence real-time cell analysis system (ACEA Biosciences, San Diego, CA, USA).

### 2.8 Organoid culture

Colon tumors from AOM/DSS mice or CRC patients were resected, cut into small pieces, and incubated in Dulbecco's Modified Eagle's Medium containing 10% FBS, 300 U/ml penicillin, 300 μg/ml streptomycin, 300 unit/ml collagenase IV and 0.2 mg/ml DNase at 37 °C for 40 min with shaking. The digested tissues were vigorously shaken to release crypts and filtered through 70-μm cell strainers. The products were centrifuged at 150 g for 5 min, then washed with cold Advanced DMEM/F12 medium twice. Thereafter, the crypts were mixed with Matrigel (Corning, NY, USA) at 1:1 ratio, plated into a 96-well plate, and incubated at 37 °C for 15 min to let the matrigel solidify. Mouse or human crypts were cultured in mouse or human IntestiCult™ Organoid Growth Medium (STEMCELL, Cambridge, MA, USA) supplemented with 100 U/ml penicillin, 100 μg/ml streptomycin for 4–5 days, and then subjected to different treatment for 24 h. The viability of organoids was measured using MTT assay as previously described [10].

### 2.9 ROS assay

Cellular ROS levels were analyzed using ROS Assay Kit (Beyotime). Adherent cells were labeled with 10 μM DCFH-DA in serum-free media at 37 °C for 20min. After trypsinzation, cells were collected and resuspended in Phosphate Buffered Saline (PBS) containing 5% FBS. Total ROS levels were analyzed using FL1 channel of the flow cytometer (Becton Dickinson, Franklin Lakes, NJ, USA).

### 2.10 C11-BODIPY 581/591 staining

C11-BODIPY 581/591 was dissolved in DMSO and diluted with 10 mM 4-(2-hydroxyethyl)-1-piperazineethanesulfonic acid (HEPES, pH = 7.2) buffer (Sigma-Aldrich). CRC cells incubated in serum-free medium were then treated with C11-BODIPY 581/591 solution at a final concentration of 5 μM at 37 °C for 30 min. After washing twice with PBS, cells were digested and re-suspended in PBS supplemented with 5% FBS, then analyzed on a flow cytometer (Becton Dickinson) using a 488 nm excitation laser.

### 2.11 GSH detection

The GSH levels in tumor cells or tissues were determined using GSH-Glo™ Glutathione Assay Kit (Promega, Madison, WI, USA) according to the manufacturer's instructions. GSH levels were normalized to the total amount of protein in cells or tissues.

### 2.12 ATAC sequencing

Butyrate- (1 mM) and PBS-treated HCT116 cells were digested and resuspended in cell cryopreservation solution, followed by immediate freezing in liquid nitrogen. The samples were sent to BioMarker Technologies (Beijing, China) for ATAC-sequencing and analysis. Briefly, the DNA libraries obtained from 50000 cells from each sample were run on a Nova6000 Illumina and clean reads were mapped to the reference genome using Bowtie2 software after filtering.

### 2.13 ChIP assay

ChIP assay was performed for butyrate-treated (1 mM, 6 h) and PBS-treated HCT116 cells using SimpleCHIP Enzymatic Chromatin IP Kit (Cell Signaling Technology, Danvers, MA, USA). According to the manufacturer's protocol, cells were crosslinked by 1% formaldehyde, neutralized with 125 mM glycine, and then harvested with ice-cold PBS containing protease inhibitor cocktail. Cell pellets were digested by micrococcal nuclease at 37 °C for 20 min, followed by sonication for 3 sets of 20 s pulses to obtain appropriate chromatin lysates. The lysates were clarified by centrifugation and then the supernatants were incubated with anti-c-FOS rabbit mAb (Cell Signaling Technology, Cat#2250T, 1:50), or rabbit IgG at 4 °C overnight. Thereafter, protein G magnetic beads were added and a 2-h incubation with rotation was conducted. After chromatin elution and DNA purification, DNA quantification was performed using SYBR green reagents on Step One real-time PCR System (Applied Biosystems,Waltham, MA, USA). The following primers were used: xCT-chip qPCR-F: GGGGTCTTTGGCTCAACTTA; xCT-chip qPCR-R: CCTCCTCCTACATCTCCTTTCA.

### 2.14 RNA-sequencing (RNA-Seq)

HCT116 cells treated with DMSO, DMSO + butyrate, erastin, erastin + butyrate were collected and resuspended in TRIZOL buffer respectively. Transcriptome sequencing was conducted using MGISEQ2000 platform by Tsingke Biotechnology Co., Ltd (Beijing, China).

### 2.15 Immunoblotting

Cells were lysed in RIPA Strong buffer (Beyotime, China) supplemented with protease and phosphatase inhibitors on ice for 30 min. After centrifugation (13000 rpm, 15 min), supernatants of protein lysates were subjected to BCA Protein Assay to determine protein concentration. Protein samples were separated by SDS-PAGE electrophoresis and transferred to PVDF membranes (Millipore, USA). The membranes were blocked with 5% BSA for 1 h, incubated with primary antibodies at 4 °C overnight, and then incubated with HRP-conjugated secondary antibodies (1:1000) for 1 h at room temperature. The immunoblots were visualized using BeyoECL Plus Kit (Beyotime, China) on Tanon Gel Imaging System (Tanon 4600, China). The primary antibodies were used as follows: xCT (Cell Signaling Technology, Cat#12691S, 1:1000), c-Fos (Cell Signaling Technology, Cat#2250, 1:2000), β-actin (HuaBio, Hangzhou, China, Cat#EM21002, 1:2000).

### 2.16 Immunohistochemistry and H-score analysis

Paraffin-embedded tissue array or xenografts were cut into consecutive 5 μm sections. The sections were rehydrated and antigen retrieval with microwave was performed. Immunohistochemistry staining was conducted using Streptavidin-HRP Rabbit & Mouse DAB Kit (CwBio, Taizhou, China). In brief, endogenous peroxydase enzyme in the sections was blocked using 0.3% H_2_O_2_ for 10 min, followed by PBS washing for 3 times. Thereafter, the sections were subjected to antigen blocking with 5% goat serum for 30 min, followed by incubation with primary antibody at 37 °C overnight. Primary antibodies were used as follows: xCT (Proteintech, Wuhan, China, Cat#26864-1-AP, 1:100), 4-HNE (R&D, Minneapolis, MN, USA, Cat#MAB3249, 1:300). On the next day, the sections were incubated with biotin-labeled secondary antibodies, streptavidin-HRP, and DAB sequentially. Then, sections were subjected to hematoxylin couterstaining for 1 min. The immunostained sections were captured with the 3D HISTECH digital Scanner (Pannoramic MIDI, Budapest, Hungary) at 400 × magnification. The xCT and 4-HNE expression levels were analyzed using the H-score method by two pathologists as previously described [11]. H-score = 1 × (% cells 1+) + 2 × (% cells 2+) + 3 × (% cells 3+).

### 2.17 Quantitative PCR

Total RNA in cells or tissues was extracted using RNA-Quick Purification Kit (YiShan Biotechnology, Shanghai, China). Then cDNA was transcribed from RNA with PrimeScript RT Master Mix (Takara, Japan). Quantitative PCR was performed using SYBR Green Mix (CwBio). The primer sequences are listed in Supplementary Table 1. The mRNA levels of target genes were normalized to β-actin mRNA.

### 2.18 Propidium iodide (PI) staining

Dead cell staining using PI was performed for cancer-stem like cells (CSCs) cultured in 96-plates. In brief, CSCs were washed with 10 mM HEPES solution once. CSCs were incubated with 25 μg/mL PI at 37 °C in the dark for 15 min. Bright field and fluorescence images were snapped for the stained CSCs using the Zeiss Axio fluorescence microscope (Oberkochen, Germany).

### 2.19 SCFA metabolomics

SCFAs in stool samples were determined by Future Co., Ltd (Qingdao, China). The sample (0.2 g), 1.3 mL 12% sulfuric acid solution, 2 mL ether, and 100 μL cyclohexanone were mixed and centrifugated to obtain the supernatant. Concentrations of SCFAs in the supernatants were determined using Agilent DB-WAX capillary column (30 m × 0.25 mm × 0.25 μm) and mass spectrum.

### 2.20 Statistical analysis

All data were expressed as mean ± standard deviation (SD). Statistical analysis was performed with GraphPad Prism 8. Unpaired Student's t-test or Spearman's rank correlation test was used where appropriate. P values of p < 0.05 were considered significant.

## 3 Results

### 3.1 Butyrate serves as a pro-ferroptotic SCFA

**Fig. 1:**
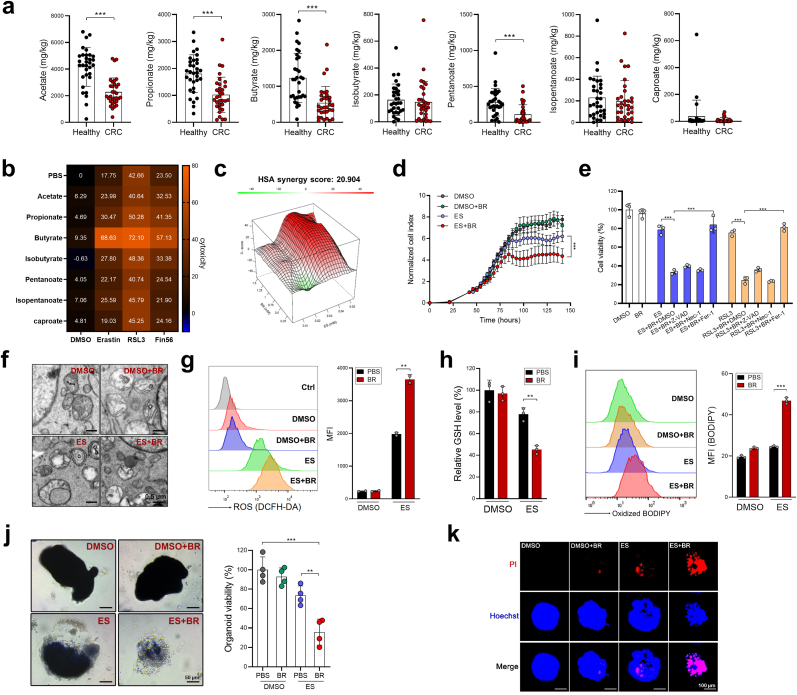
Butyrate enhances ferroptosis sensitivity in CRC cells. **a,** Fecal levels of SCFAs in CRC patients (*n* = 32) and healthy controls (*n* = 32) were evaluated by SCFA metabolomics (for isobutyrate or caproate detection, *n* = 31 or 25 respectively, since some patients had low levels of isobutyrate or caproate which were below the detection threshold). **b,** HCT116 cells were pretreated with different SCFAs (1 mM) for 8 h, followed by treatment of erastin (30 μM), RSL3 (15 μM), FIN56 (5 μM) or DMSO for 24 h. Cell viability was evaluated by CCK8 assay. **c,** The synergistic effects of butyrate and erastin were assessed by the SynergyFinder tool. **d,** HCT116 cells were treated with butyrate, erastin, or combination and cell viability was evaluated by a RTCA system. Normalized Cell Index means that in each group, the cell indexes were normalized to the corresponding cell index of the time point of adding erastin. **e,** HCT116 cells were pretreated with butyrate (1 mM) for 8 h in the presence of Z-VAD-FMK (10 μM), ferrostatin-1 (Fer-1, 2 μM), or necrostatin-1 (Nec-1, 2 μM), followed by the treatment of erastin for 24 h. Cell viability was evaluated by CCK8 assay. **f-i** HCT116 cells were pretreated with butyrate (1 mM) followed by erastin (20 μM) treatment. Mitochondrial morphology was evaluated by transmission electron microscope (**f**). The intracellular ROS production (**g**), GSH levels (**h**) and lipid peroxidation (**i**) were measured. MFI = mean fluorescence intensity. **j, k** CRC organoids from AOM/DSS mice were treated with butyrate, erastin, or combination. Organoid viability was measured by MTT assay (**j**) and PI staining **(k**). Representative images (left) and quantification data (right) are shown. Data are represented as the mean ± SD. **p < 0.01; ***p < 0.001, two-tailed unpaired Student's *t*-test.

First, we tested the sensitivity of cell lines from 20 types of human cancers to erastin (a typical ferroptosis inducer) using the DepMap database (https://depmap.org/portal/). The results indicated that CRC is the forth most insensitive tumor type (Supplementary Fig. S1) to erastin, suggesting that CRC is relatively resistant to ferroptosis induction. SCFAs are the most abundant microbial fermentation products in the colon [4], by performing SCFA metabolomics, we discovered that among the seven major SCFAs, the levels of acetate, propionate, butyrate, pentanoate, were markedly downregulated in the stools from CRC patients compared to those from healthy controls (Fig. 1a). We then investigated if SCFAs influenced CRC cell ferroptosis through *in vitro* screening. As shown in Fig. 1b, among all the seven SCFAs, butyrate treatment dramatically sensitized HCT116 cells to ferroptosis-inducing agents (FINs), including erastin (type I FIN), RSL3 (type I FIN) and Fin56 (type III FIN), while the other SCFAs exhibited mild to no effects. The synergistic effect of butyrate and erastin was further evaluated by adopting a computational tool, SynergyFinder, which was designed for calculating the combinatory effect of two drugs [12]. Computational modeling revealed a high synergy score of 20.904 (Fig. 1c). Using a Real Time Cell Analysis (RTCA) system, we found that erastin reduced HCT116 cell viability more efficaciously in the presence of butyrate over time (Fig. 1d). The physiological range of butyrate concentration in mouse distal colon was reported to be approximately 0.5–1 mM [13]. Therefore, this dose range of butyrate was used in our present work unless otherwise stated. The synergistic effect of butyrate and erastin or RSL3 could be reversed by the ferroptosis inhibitor ferrostatin-1, but not by the apoptosis inhibitor (Z-VAD-FMK), nor the necrosis inhibitor (necrostatin-1) (Fig. 1e), alluding to the specific pro-ferroptotic properties of butyrate. Although erastin treatment alone induced only mild degrees of mitochondrial damage, ROS production, GSH deprivation, and lipid peroxidation, the combinatory effect of erastin and butyrate resulted in a marked increase of these ferroptotic phenotypes (Fig. 1f–i). In addition to HCT116 cells, which carry *KRAS*^G13D^*PIK3CA*^H1047R^ mutation, the pro-ferroptotic effect of butyrate was also observed in RKO cells (*BRAF*^V600E^*PIK3CA*^H1047R^), SW480 and SW620 cells (both are *KRAS*^G12V^*TP53*^R273H;P309S^) (Supplementary Fig. S2), suggesting that butyrate sensitizes ferroptosis irrespective of the common oncogenic mutations. To test this synergistic effect further, we further validated that butyrate markedly increased the cytotoxicity of erastin to mouse CRC organoids (Fig. 1j and k). Therefore, we are confident that butyrate could sensitize CRC cells to ferroptosis and that would translate to *in vivo* models.

### 3.2 Butyrate enhances the therapeutic efficacy of ferroptosis in CRC models

**Fig. 2:**
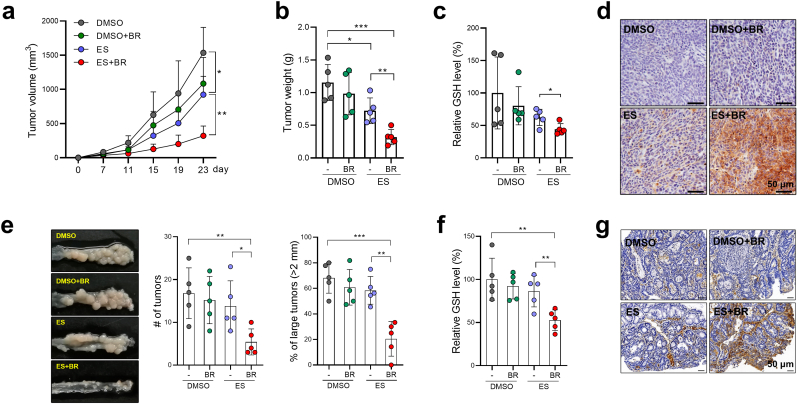
Butyrate supplementation enhances tumor susceptibility to ferroptosis in CRC mice. **a,** HCT116-bearing mice were administered butyrate, erastin, or both (*n* = 5/group). Tumor volume was monitored. **b,** Tumors were photographed and weighed at the experimental endpoint (Day 23). **c,** Tumor GSH levels were evaluated. **d,** Lipid peroxidation was evaluated by 4-HNE staining. **e-g** AOM/DSS-CRC mice were administered with butyrate, erastin, or both (*n* = 5/group). The size and number of tumor nodules were measured (**e**). (**f**) The levels of GSH in tumor tissues were evaluated. **(g**) The lipid peroxidation levels in tumor tissues were evaluated by 4-HNE staining. Data are represented as the mean ± SD. *p < 0.05; **p < 0.01; ***p < 0.001, two-tailed unpaired Student's *t*-test.

To explore the potential role of butyrate in CRC ferroptosis, we established two CRC models. First, we inoculated HCT116 cells s.c. into nude mice, which were then treated with either erastin alone or a combination of erastin and butyrate. Erastin monotherapy led to a moderate delay in tumor growth (Fig. 2a and b). Strikingly, the combination of erastin and butyrate drastically inhibited tumor growth by ∼80% which was exhibited by the reduced size and weight (Fig. 2a and b). Similarly, butyrate supplementation also significantly improved the anti-tumor effect of erastin in a SW480 CRC model (Supplementary Fig. S3).

In line with the increased ferroptosis, GSH levels were significantly reduced in butyrate + erastin treated tumors compared to those in the erastin only control group (Fig. 2c). 4-hydroxy-2-noneal (4-HNE) staining also revealed that lipid peroxidation was greatly increased in butyrate + erastin treated tumors, whereas erastin monotherapy failed to induce obvious lipid peroxidation (Fig. 2d).

We then generated an AOM/DSS model in which colon tumors were developed *in situ* driven by AOM-induced DNA damage followed by chronic intestinal inflammation [14]. The co-administration of erastin and butyrate, but not erastin administration alone, markedly reduced the average size and the number of tumor nodules in the distal colon (Fig. 2e). In addition, combinatory treatment with butyrate and erastin also reduced GSH levels and increased lipid peroxidation in tumors (Fig. 2f and g). Hence, the anti-tumor efficacy of ferroptosis inducers is boosted when used in combination with butyrate.

### 3.3 Butyrate suppresses the expression of xCT by inhibiting HDAC activity

**Fig. 3:**
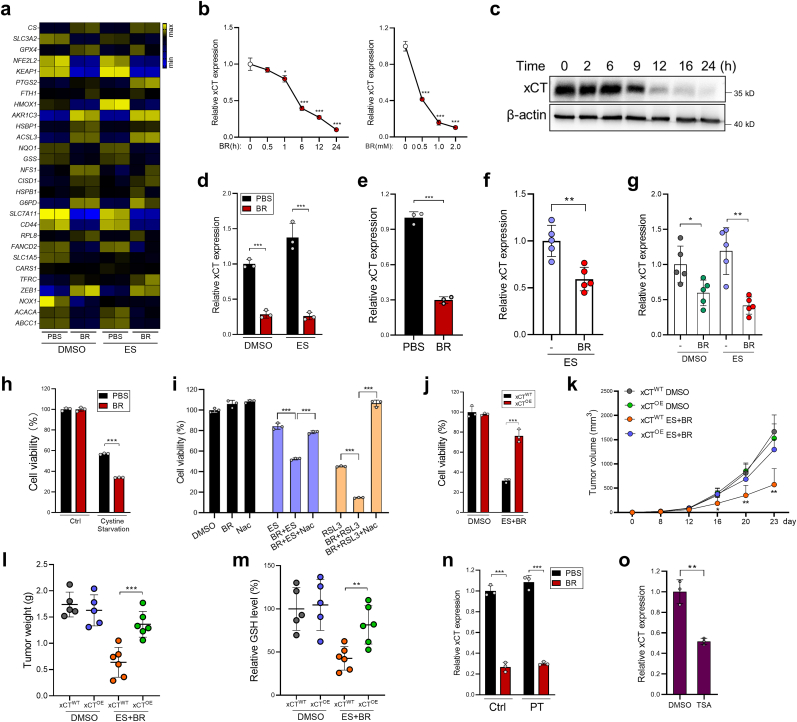
Butyrate inhibits xCT expression via HDAC inhibition. **a,** HCT116 cells were treated with DMSO or erastin (20 μM) for 10 h in the presence or absence of butyrate (1 mM), then subjected to RNA-Seq. The expression levels of ferroptosis–related genes were shown. **b,c** HCT116 cells were treated with butyrate as indicated, xCT expression was evaluated by qPCR (**b**) and immunoblotting (**c**). **d,** Mouse CRC organoids were treated with butyrate; xCT expression was evaluated by qPCR. **e,** Organoids from CRC patients were stimulated with 1 mM butyrate for 12 h, xCT expression was evaluated by qPCR. **f, g** xCT expression in HCT116 tumors (**f)** and AOM/DSS tumors (**g)** were evaluated by qPCR. **h,** HCT116 cells treated with butyrate were cultured in cystine-sufficient or cystine-low conditions. Cell viability was evaluated by CCK8. **i,** HCT116 cells were treated with erastin (20 μM) or RSL3 (10 μM), either alone or in combination with butyrate in the absence or presence of NAC. Cell viability was evaluated by CCK8. **j,** xCT^OE^ and control HCT116 cells were treated with a combination of butyrate + erastin. Cell viability was evaluated by CCK8. **k,** xCT^OE^ or control HCT116 cells were inoculated s.c into nude mice, which were then treated with DMSO or butyrate + erastin (*n* = 5–6/group). Tumor growth was monitored. **l,** On day 23 mice were sacrificed and tumors were weighed. **m,** Tumor GSH levels were measured. **n,** HCT116 cells were pretreated with pertussis toxin (PT) for 2 h, followed by butyrate treatment for 12 h xCT expression was evaluated by qPCR. **o,** HCT116 cells were treated with TSA (10 mM). xCT expression was evaluated by qPCR. Data are represented as the mean ± SD. *p < 0.05; **p < 0.01; ***p < 0.001, two-tailed unpaired Student's *t*-test.

To explore the mechanism of action of butyrate, RNA-Seq was performed. Among the known ferroptosis-related genes, butyrate treatment decreased the expression of xCT (coded by *SLC7A11*) by approximately 80% in both DMSO- or erastin-treated HCT116 cells (Fig. 3a). Through qPCR and immunoblotting, we further confirmed that butyrate dose- and time-dependently reduced xCT expression in CRC cells (Fig. 3b and c). To confirm that reduced xCT expression is a butyrate dependent mechanism, we validated that other SCFAs have moderate or no effect (Supplementary Fig. S4). Also, organoids from CRC mice or CRC patients treated with butyrate showed significantly reduced xCT expression (Fig. 3d and e). Consistently, butyrate administration significantly reduced tumor expression of xCT in aforementioned CRC models (Fig. 3f and g). Furthermore, juxtaposed with HCT116 cells cultured under cystine-enriched conditions, butyrate treatment significantly reduced the viability of HCT116 cells cultured under cystine-deficient conditions (Fig. 3h). In contrast, the pro-ferroptotic effect of butyrate was abrogated by *N*-acetylcysteine (NAC), a common antioxidant, which can be transported into cells and contribute to GSH synthesis independent of xCT function, indicating the inhibitory role of butyrate on cystine transport (Fig. 3i).

We then generated stable xCT-overexpressed HCT116 cells (xCT^OE^) and discovered xCT overexpression drastically abrogated ferroptosis of HCT116 cells after treatment with butyrate + erastin (Fig. 3j). Moreover, compared to control HCT116 cells, mice inoculated with xCT^OE^ cells were resistant to butyrate + erastin treatment, evidenced by both accelerated tumor growth and increased tumor GSH levels (Fig. 3k-m).

Butyrate has been reported to regulate cell functions by two key pathways [15]: 1) activation of GPCR signaling [16,17], 2) regulation of chromatin configuration through HDAC inhibition (HDACi) [18,19]. Pretreatment of HCT116 cells with pertussis toxin (a GPCR signaling inhibitor) failed to reverse the effects of butyrate on xCT expression (Fig. 3n). Conversely, Trichostatin A (TSA), a pan-HDAC inhibitor, recapitulated the xCT-suppressive function of butyrate (Fig. 3o). Further analysis revealed that among the five HDAC subtypes, the inhibition of class I HDACs (HDAC1/2/3) by entinostat exhibited the strongest xCT-inhibitory and pro-ferroptotic effects, whereas the inhibition of other HDAC classes had either minor effect (class IIa HDACs) or no effect (class IIb, III, IV HDACs) (Supplementary Figs. S5a and b). These results indicate that butyrate suppresses xCT expression via inhibiting class I HDAC activity.

### 3.4 Butyrate level correlates with tumor xCT expression in CRC patients

**Fig. 4:**
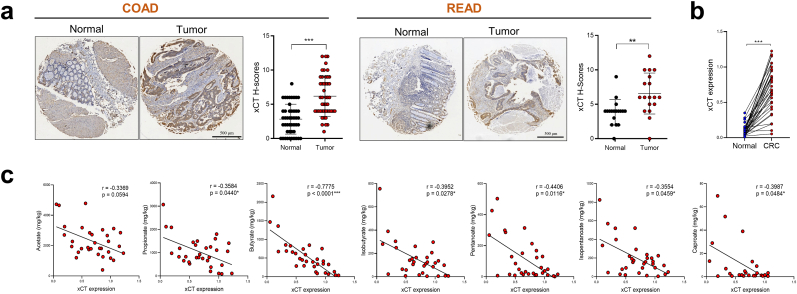
Low butyrate concentration is correlated with high xCT expression in CRC patients. **a, b** The levels of xCT protein (**a**) or mRNA (**b**) in CRC tissues and adjacent-normal tissues were evaluated by tissue microarrays (*n* = 47 for COAD patients, *n* = 18 for READ patients) or qPCR (n = 32/group), respectively. COAD = colon adenocarcinoma, READ = rectum adenocarcinoma. **c,** Correlations between tumor xCT mRNA expression and fecal SCFA concentration were analyzed by Spearman's rank correlation test (*n* = 32. For isobutyrate or caproate detection, *n* = 31 or 25 respectively. Since some patients have low levels of isobutyrate or caproate which were below the detection threshold). Data are represented as the mean ± SD. *p < 0.05; **p < 0.01; ***p < 0.001, two-tailed unpaired (**a**) or paired (**b**) Student's *t*-test.

Given the promising *in* vivo efficacy of butyrate on suppressing xCT expression, we next hypothesized that this effect would be potentially clinically relevant. Through qPCR and tissue microarray, we discovered that xCT levels were significantly increased in CRC tissues compared with that in the matched tumor-adjacent tissues (Fig. 4a and b). This upregulation of xCT might represent a compensatory mechanism against the high oxidative stress in tumor tissues compared with the adjacent normal tissues from the same patients. Importantly, a significant negative correlation between fecal butyrate concentration and tumor xCT level was observed in CRC patients (Fig. 4c), indicating that butyrate production is a key determinant of xCT expression and ferroptosis sensitivity in CRC patients.

### 3.5 Butyrate suppresses xCT-mediated ferroptosis resistance by inducing c-Fos expression

**Fig. 5:**
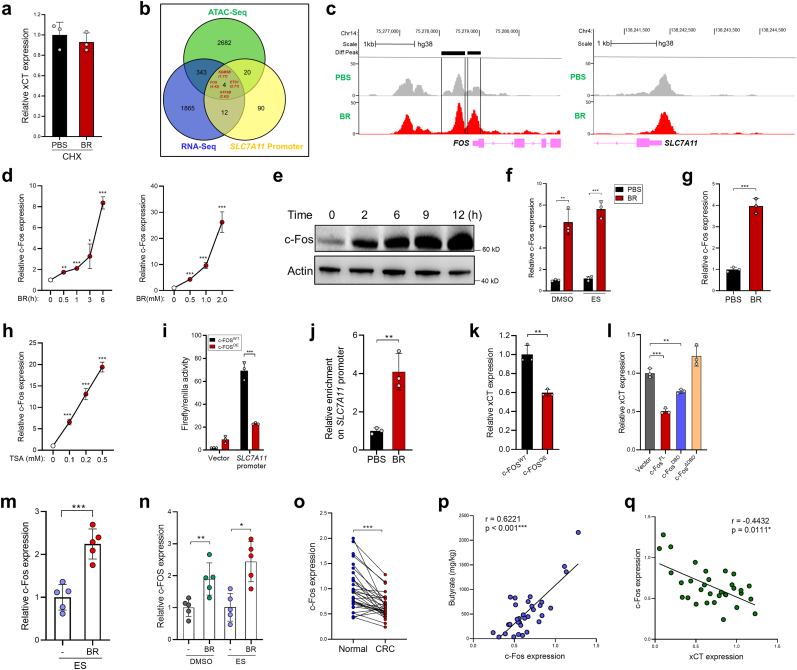
Butyrate induces c-Fos expression in CRC cells. **a,** HCT116 cells were treated with PBS or butyrate (1 mM) for 12 h in the presence of CHX (5 μg/ml), xCT expression was evaluated by qPCR. **b,** HCT116 cells were treated with 1 mM butyrate for 1 h. The genes having increased chromatin accessibilities (fold change >1.5) in their promoter region were overlapped with the butyrate-upregulated genes from RNA-Seq data, and with the genes whose products were predicted to bind to *SLC7A11* promoter using hTFtarget tool (http://bioinfo.life.hust.edu.cn/hTFtarget#!/). **c,** Differential peaks of ATAC-seq signal in PBS- or butyrate-treated HCT116 cells at *FOS* and *SLC7A11* promoter region. **d, e** HCT116 cells were treated with butyrate as indicated and c-Fos expression was evaluated by qPCR (**d**) and immunoblotting (**e**). **f,** Mouse CRC organoids were treated with butyrate, erastin, or both and c-Fos expression was evaluated by qPCR. **g,** Organoids from CRC patients were stimulated with 1 mM butyrate, c-Fos expression was evaluated by qPCR. **h,** HCT116 cells were treated with TSA and c-Fos expression was evaluated by qPCR. **i,** C-Fos was overexpressed in HCT116 cells, the activity of the *SLC7A11* promoter was evaluated by a dual-luciferase reporter assay. **j,** The binding of c-Fos to the *SLC7A11* promoter was examined by a ChIP assay. **k,** The expression of xCT was evaluated by qPCR after c-Fos overexpression. **l,** Different c-Fos protein domains were ectopically expressed in HCT116 cells. The expression of xCT was evaluated by qPCR. **m, n** C-Fos expression in HCT116 tumors (**m**) and AOM/DSS tumors (**n**) were evaluated by qPCR. **o,** The mRNA expression of c-Fos in CRC tissues and adjacent-normal tissues were evaluated by qPCR. **p,** The correlation between tumor c-Fos mRNA expression and fecal butyrate concentrations was analyzed by Spearman's rank correlation test (*n* = 32). **q,** The correlation between tumor c-Fos expression and xCT expression was analyzed by Spearman's rank correlation test (*n* = 32). Data are represented as the mean ± SD. *p < 0.05; **p < 0.01; ***p < 0.001, two-tailed unpaired (**a-n**) or paired (**o**) Student's *t*-test.

HDAC inhibition facilitates the opening of chromatin which leads to transcriptional activation; therefore it is unlikely that butyrate directly inhibits xCT through its HDACi activity. Indeed, butyrate failed to suppress xCT expression in CRCs cells exposed to cycloheximide (Fig. 5a), indicating that butyrate downregulated xCT expression by triggering the *de novo* protein synthesis of a potential xCT suppressor. Therefore, we performed Assay for Transposase Accessible Chromatin with high-throughput sequencing (ATAC-seq) to globally examine how butyrate modulates chromatin accessibility in CRC cells. The results revealed that 1 h after butyrate treatment, 3049 genes exhibited enhanced chromatin accessibilities (fold change >1.5) in their promoter region. We then overlapped the 3049 genes having increased promoter chromatin accessibilities with the butyrate-upregulated genes in our RNA-Seq data, and with the genes whose products were predicted to bind to *SLC7A11* promoter. Four genes were found in the intersection of the three datasets, with *FOS* (c-Fos encoding gene) showing the most robust expression upregulation by butyrate (4.42 fold) (Fig. 5 b, c). Interestingly, although butyrate acts as a HDACi, we identified 3831 genes whose promoter chromatin accessibilities were reduced in butyrate-treated HCT116 cells. However, the chromatin accessibility in *SLC7A11* promoter was not obviously altered by 1-h butyrate treatment (Fig. 5c), suggesting that butyrate-induced xCT suppression might be a late secondary effect.

Consistent with the ATAC-Seq and RNA-seq data, we further confirmed that butyrate upregulated c-Fos expression in CRC cells (Fig. 5d and e), as well as in organoids from both CRC mice and CRC patients (Fig. 5f and g). TSA treatment recapitulated the effect of butyrate (Fig. 5h). A dual-luciferase reporter assay showed that the activity of xCT promoter was significantly decreased in c-Fos overexpressed (c-Fos^OE^) HCT116 cells, compared to that in control HCT116 cells (Fig. 5i), suggesting that c-Fos transcriptionally suppresses xCT expression. By performing Chromatin Immunoprecipitation (ChIP) assay, we verified that butyrate treatment induced the binding of c-Fos to the xCT promoter region (Fig. 5j). Furthermore, overexpression of c-Fos in HCT116 cells (c-Fos^OE^) reduced xCT expression (Fig. 5k).

To clarify the protein domain required for the c-Fos-mediated xCT inhibition, full-length c-Fos (c-Fos^FL^), the DNA-binding domain of c-Fos (c-Fos^DBD^), and c-Fos lacking the DNA-binding domain (c-Fos^ΔDBD^) were ectopically expressed in HCT116 cells separately. Compared with c-Fos^FL^, c-Fos^DBD^ partially reduced xCT expression, while c-Fos^ΔDBD^ completely eradicated this function (Fig. 5l), suggesting that the DNA-binding capacity of c-Fos is necessary, but not sufficient for the c-Fos-mediated xCT inhibition.

We then translated this to *in vivo* experiments and further demonstrated that butyrate administration significantly increased c-Fos expression in HCT116 tumors and AOM/DSS tumors (Fig. 5m and n). In CRC patients, the expression of c-Fos was significantly lower in tumor tissues compared with that in the adjacent normal tissues (Fig. 5o). Importantly, the level of tumor c-Fos expression was positively correlated with fecal butyrate level (Fig. 5p), yet negatively correlated with xCT expression in CRC patients (Fig. 5q).

**Fig. 6:**
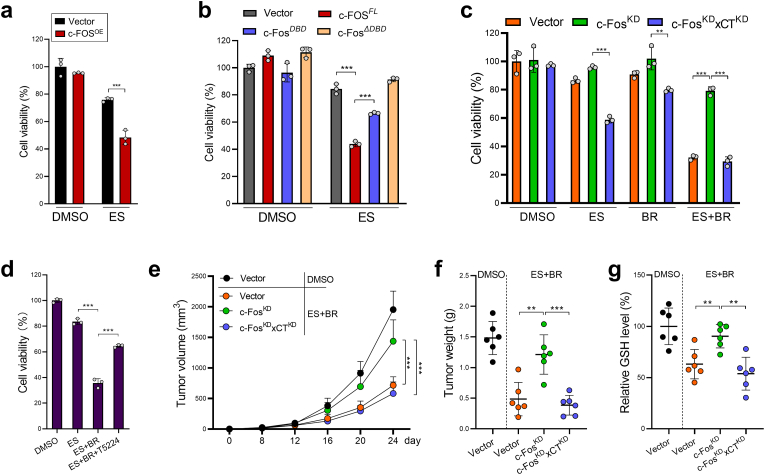
Butyrate treatment breaks xCT-mediated ferroptosis resistance in a c-Fos-dependent manner. **a,** Control or c-Fos^OE^ HCT116 cells were treated with erastin for 24 h. Cell viability was evaluated by CCK8. **b,** Different c-Fos protein domains were ectopically expressed in HCT116 cells followed by erastin treatment for 24 h. Cell viability was evaluated by CCK8. **c,** Control, c-Fos^KD^, or c-Fos^KD^xCT^KD^ HCT116 cells were treated with erastin, butyrate, or both for 24 h. Cell viability was evaluated by CCK8. **d,** Mouse CRC organoids were treated with butyrate + erastin in the presence of a c-Fos inhibitor (T5224, 20 μM) for 24 h. Organoid viability was evaluated by MTT. **e,** Control, c-Fos^KD^, or c-Fos^KD^xCT^KD^ HCT116 cells were inoculated s.c. into nude mice, which were then treated with butyrate + erastin. Tumor growth was monitored. **f,** Mice were sacrificed on day 24 and tumors were weighed. **g,** Tumor GSH levels were evaluated. Data are represented as the mean ± SD. *p < 0.05; **p < 0.01; ***p < 0.001, two-tailed unpaired Student's *t*-test.

Our cell viability assay demonstrated that c-Fos overexpression significantly sensitized HCT116 cells to erastin-induced ferroptosis (Fig. 6a). Furthermore, loss of the DNA-binding domain entirely abrogated the pro-ferroptotic effect of c-Fos (Fig. 6b). In contrast, c-Fos knockdown (c-Fos^KD^) significantly abrogated the pro-ferroptotic role of butyrate, while this effect was reversed by the concomitant knockdown of xCT (c-Fos^KD^xCT^KD^) (Fig. 6c). Moreover, treatment of T5224 (a c-Fos inhibitor) rescued the viability of butyrate + erastin challenged CRC organoids (Fig. 6d). In nude mice, c-Fos^KD^ HCT116 cells developed significantly larger tumors after butyrate + erastin combinatory treatment in comparison to control HCT116 cells; however, tumor growth was significantly reduced by the concomitant knockdown of xCT (c-Fos^KD^xCT^KD^) (Fig. 6e–f). Consistent with the tumor growth data, c-Fos^KD^ tumors had significantly higher GSH level than both control and c-Fos^KD^xCT^KD^ tumors (Fig. 6g). Thus, the pro-ferroptotic function of butyrate is dependent on c-Fos-mediated xCT suppression.

### 3.6 Butyrate breaks ferroptosis resistance of cancer stem cells

**Fig. 7:**
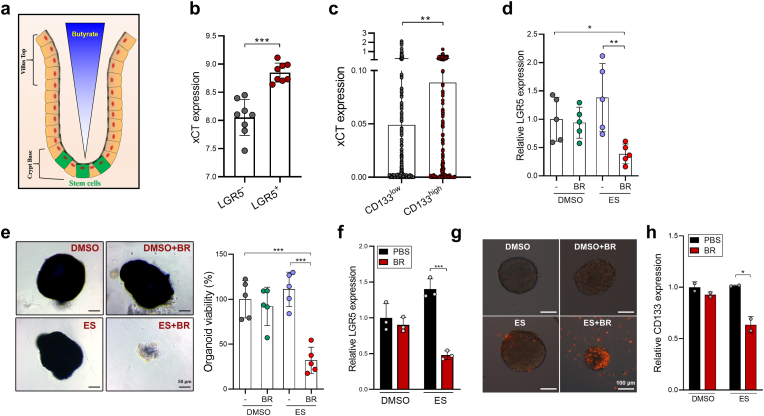
Butyrate reduces the ferroptosis resistance of CSCs. **a,** The luminal-to-crypt gradient of butyrate in the large intestine. **b,** The expression of xCT in LGR5^+^ and LGR5^-^ non-CSCs were evaluated by analyzing a GEO dataset (GSE92961). **c,** The expression of xCT in CD133^+^ CSCs and CD133^-^ non-CSCs in CRC patients were evaluated by analyzing a single-cell sequencing dataset (GSE146771). **d,** The expression of LGR5 was evaluated in AOM/DSS CRC tissues by qPCR. **e,** The viability of colon organoids isolated from AOM/DSS CRC mice administered with butyrate, erastin or in combination were evaluated by MTT assay. **f,** CRC organoids were treated with erastin (10 μM), butyrate (0.5 mM), or both for 24 h. The expression of LGR5 was evaluated by qPCR. **g,** HCT116 CSC spheres were treated with erastin (20 μM), butyrate (0.5 mM), or both for 24 h. The viability was examined by PI staining. **h,** The expression of CD133 was evaluated by qPCR. Data are represented as the mean ± SD. *p < 0.05; **p < 0.01; ***p < 0.001, two-tailed unpaired Student's *t*-test.

The colonic epithelium has a special architecture wherein the colonocytes at the top of crypts can consume butyrate and thus protect crypt base stem cells from butyrate exposure [13,20] (Fig. 7a). Indeed, we found higher xCT expression in LGR5^+^ colorectal stem cells (CSCs) than those in LGR5^-^ non-CSC population in a published GEO dataset (GSE92961) (Fig. 7b). Through exploring a human CRC single-cell sequencing dataset (GSE146771), we also demonstrated that CD133^+^ CSCs expressed significantly higher level of xCT compared to CD133^-^ non-CSCs (Fig. 7c), indicating that CSCs might be more resistant to ferroptosis.

Interestingly, although erastin treatment reduced tumor progression in AOM/DSS mice (Fig. 2e), these mice exhibited increased expression of CSC markers (e.g. LGR5) compared with control mice, suggesting that erastin induces lower cytoxicity to CSCs than non-CSCs (Fig. 7d). Strikingly, erastin treatment combined with butyrate supplementation significantly reduced tumor expression of LGR5 (Fig. 7d). Furthermore, colon organoids derived from butyrate + erastin treated-, but not those from erastin alone-treated mice had markedly impaired growth potential, indicating that butyrate effectively breaks the resistance of CSCs to ferroptosis (Fig. 7e). Consistent with the *in vivo* data, although erastin challenge impaired the viability of CRC organoids (Fig. 1j), the expression of LGR5 was mildly increased (Fig. 7f). In contrast, combinatory treatment with erastin + butyrate led to a dramatic reduction of LGR5 expression in CRC organoids.

We then cultured HCT116 CSC spheres followed by erastin treatment. Again, the combination of butyrate and erastin, but not erastin alone, effectively reduced the viability and the level of CD133 in tumor spheres (Fig. 7g and h). Therefore, butyrate supplementation preferentially increases the ferrosensitivity of cancer stem cells, this might help prevent tumor recurrent after ferroptosis induction.

### 3.7 Butyrate potentiates the pro-ferroptotic function of oxaliplatin

**Fig. 8:**
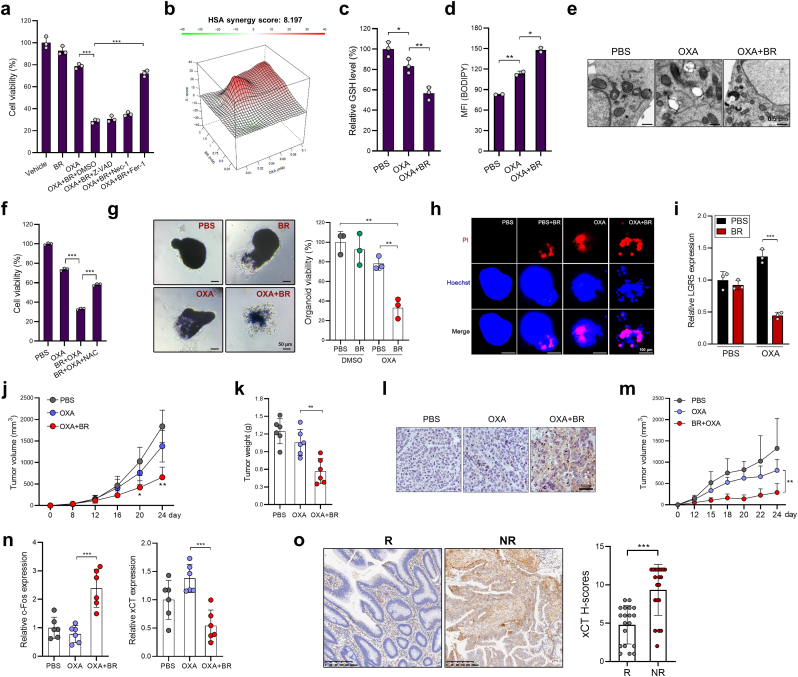
Butyrate potentiates the therapeutic sensitivity of OXA. **a,** HCT116 cells were treated with OXA (20 μM), butyrate (1 mM), or both for 36 h, in the presence or absence of Z-VAD, nec-1, or fer-1. Cell viability was evaluated by CCK8. **b,** The synergistic effects of butyrate and OXA were assessed by SynergyFinder. **c-e** HCT116 cells were treated as indicated for 36 h, GSH levels **(c)**, lipid peroxidation (**d**), and mitochondria morphology (**e**) were evaluated. **f,** HCT116 cells were treated as indicated for 36 h, cell viability was evaluated by CCK8. **g, h** CRC organoids were treated with OXA, butyrate, or both. Organoid viability was determined by MTT (**g**) and PI staining (**h**). **i,** The expression of LGR5 was evaluated by qPCR. **j-l** HCT116 cells were inoculated s.c. into nude mice, which were then treated with OXA or a combination of OXA plus butyrate (*n* = 6/group). Tumor growth was monitored (**j**), tumor weight was evaluated on day 24 (**k**), 4-HNE staining was performed to identify lipid peroxidation (**l**). **m,** Hep3b cells were inoculated s.c. into nude mice, which were then treated with OXA or OXA plus butyrate (*n* = 5/group). Tumor growth was monitored. **n,** The expression of xCT and c-Fos in HCT116 tumor tissues were evaluated by qPCR. **o,** The expression of xCT in OXA responders (R, *n* = 19) and non-responders (NR, *n* = 19) was examined by IHC. Data are represented as the mean ± SD. *p < 0.05; **p < 0.01; ***p < 0.001, two-tailed unpaired Student's *t*-test.

Subsequently, we sought to explore the clinical relevance of butyrate-mediated suppression of xCT. Previous reports have revealed that certain traditional anti-cancer drugs, such as cetuximab, gemcitabine, or paclitaxel, can serve as ferroptosis inducers or sensitizers [[21], [22], [23]]. We thus deliberated if a first-line chemotherapy drug for CRC - oxaliplatin (OXA), can also induce ferroptotic cell death. HCT116 cells, a known OXA-resistant cell line, were significantly more susceptible to ferroptosis induction when treated in combination with butyrate (Fig. 8a). Importantly, this phenomenon was reversed by ferrostatin-1 but not by Z-VAD-FMK or necrostatin-1 (Fig. 8a), confirming a ferroptotic specific cell death pathway. Using the computational tool SynergyFinder, OXA and butyrate were found to have a relatively high synergy score (8.197) (Fig. 8b). Moreover, OXA marginally downregulated the GSH level, enhanced lipid peroxidation and mitochondrial damage in HCT116 cells, all these three cellular responses were exacerbated upon sensitization with butyrate (Fig. 8c–e). GSH supplementation prevented the death of OXA + butyrate-cotreated HCT116 cells (Fig. 8f), indicating that butyrate bolsters OXA-induced ferroptosis by GSH deprivation. In addition, butyrate significantly enhanced the cytotoxicity of OXA to CRC organoids, and significantly reduced LGR5 expression in CRC organoids (Fig. 8g–i).

In HCT116 tumor-bearing mice, co-administration of OXA and butyrate effectively delayed tumor growth and increased lipid peroxidation (Fig. 8j-l), meanwhile OXA monotherapy only marginally inhibited tumor development. The similar effect was also observed in Hep3b tumor-bearing mice - a liver cancer model (Fig. 8m), suggesting that butyrate might also improve the response to OXA in liver cancer patients [24]. Moreover, butyrate administration suppressed xCT expression, while increasing c-Fos expression in OXA-treated mice (Fig. 8n). Finally, we investigate whether ferroptosis sensitivity correlates with OXA resistance. Through IHC, we found that tumor tissues from OXA responders had significantly lower xCT protein expression when compared to OXA non-responders (Fig. 8o), indicating that suppressing xCT expression could pose as a critical link in overcoming OXA resistance.

In conclusion, butyrate breaks the resistances of CRC, especially cancer stem cells to ferroptosis by inducing c-Fos dependent xCT inhibition.

## 4 Discussion

SCFAs have been long considered to act as beneficial metabolites that maintain gut homeostasis through diverse mechanisms [[25], [26], [27]]. In recent years, emerging evidence has revealed the impacts of SCFAs on cancer therapy. For example, systemic SCFAs were reported to limit the effect of anti-CTLA-4 tumor immunotherapy in melanoma patients [5]. Moreover, pentanoate and butyrate enhanced function of anti-tumor chimeric antigen receptor (CAR) T cells in melanoma and pancreatic cancer [6]. In addition, butyrate treatment increased the efficacy of radiotherapy in CRC [8]. Accordingly, the exact role of SCFAs, in this specific case butyrate, on cancer therapy is complex and is dependent upon the treatment strategy, cancer type, and other complex biological factors.

In this work, we present the impacts of SCFAs on CRC ferroptosis and revealed butyrate as a pro-ferroptotic SCFA. As a new form of cell death, ferroptosis is relatively less affected by the oncogenic mutations commonly seen in human cancers such as *KRAS*, *TP53*, or *BRAF* [28], which are considered heavily pro-tumorigenic. Despite this, cancer cells also evolve many mechanisms to antagonize ferroptotic death. Among those, xCT-mediated cystine transport is of particular importance. According to our bio-informatic analyses, CRC cell lines are relatively insensitive to ferroptosis. Herein, we elucidate that the reduced butyrate concentration in CRC patients is the major contributing factor for the increased xCT expression, ultimately driving the resistance of CRC to ferroptosis.

Compared to the relatively high butyrate dose in the proximal colon (∼3–5 mM), the physiological level of butyrate in the distal colon was shown to be below 1 mM in mice [13]. Actually, at an ultra-physiological concentration (>10 mM), butyrate alone caused prominent cell death which was partially reversed by ferrostatin-1 and NAC (data not shown). Although the concentration of butyrate is high in mouse or human fecal, obviously, butyrate did not exhibit cytotoxic effect to IECs *in vivo* at the physiological condition [29,30], even after exogenous supplementation in drinking water (data not shown). Therefore, in our *in vitro* experiments we used a butyrate dose ranging from 0.5 mM to 1 mM which is insufficient to induce obvious CRC cell death. Instead, it specifically sensitizes CRC cells to ferroptosis, yet not apoptosis or necrosis. Furthermore, our present work also highlights that due to the luminal-to-crypt gradient of butyrate [13,20], crypt base CSCs retained higher resistance to ferroptosis, which could be effectively overcome by butyrate supplementation. Intriguingly, our RNA-Seq result showed that butyrate-treated HCT116 cells had markedly decreased expression of CD44 (data not shown), which is required for stabilizing xCT on cell membrane [31]. This might provide another explanation for preferential effect of butyrate on CSCs, since they express higher levels of CD44 than normal tumor cells [32]. Therefore, in CSCs, butyrate could suppress both CD44 and xCT expression, and thus disrupts the xCT/CD44 complex, generating an additional mechanism apart from transcriptional downregulation of xCT.

Mechanistically, although SCFAs are known to regulate cell functions via binding to GPCR [17,33], blocking GPCR signaling by PT failed to abrogate the function of butyrate in our work. Instead, the pro-ferroptotic capacity of butyrate mainly relies on c-Fos induction through HDAC inhibition. In some cancers, c-Fos was found to be overexpressed and promotes tumor progression. However, the anti-tumor functions of c-Fos were also reported. High c-Fos expression has been associated with better prognosis in breast cancer, gastric cancer and ovarian cancer patients [34,35]. Besides, c-Fos is known to suppress hepatocellular carcinoma and colorectal carcinoma by inducing tumor cell apoptosis [36,37]. Moreover, c-Fos inhibits ovarian cancer progression by reducing tumor cell adhesion to peritoneal surfaces [38]. In the present work, we observed that c-Fos expression was downregulated in CRC patients and is positively correlated with patients’ fecal butyrate levels. As a component of AP-1 transcription factor, binding of the c-Fos to gene promoter is usually associated with transcriptional activation; however, in certain circumstances, c-Fos can function as a transcriptional repressor as well. For example, c-Fos downregulates the expression of TNF-α, IL-6 and IL-12 in macrophages through inhibiting NF-κB activity [39,40]. In myocytes, c-Fos suppressed the promoter activity of Atrial Natriuretic Factor (ANF) gene [41]. Up until now the mechanisms responsible for the c-Fos-mediated transcription repression have never been investigated. In our work, we revealed that DNA-binding capacity of c-Fos is necessary, but not sufficient for the c-Fos-mediated xCT inhibition. It is possible that c-Fos occupies the binding sites of certain transcription factors that activate xCT expression, or c-Fos can directly serve as a key component of a transcriptional repressive complex. The exact molecular mechanism still needs further investigation.

From a therapeutic perspective, compared to other previously identified xCT suppressors (ATF3 or p53), butyrate is a more attractive alternative given its higher practical value as an endogenous metabolite, which can be delivered by many means including as oral administration, fecal microbiota transplantation (FMT), or supplementation of butyrate-producing bacteria (e.g. *Lactobacillus rhamnosu*s GG) [[42], [43], [44]]. Notably, the higher levels of xCT in OXA-nonresponders than OXA-responders indicated that suppressing xCT expression might potentiate the responsiveness of OXA treatment. This is in agreement with previous reports that xCT expression contributes to platinum resistance in cancer patients [45,46]. Therefore, our work also highlights that fecal butyrate levels could serve as a potential biomarker in predicting the therapeutic outcome of anti-tumor drugs which induce ferroptotic cell death.

## 5 Conclusions

The reduced production of butyrate in CRC patients is a crucial factor for the insensitivity of ferroptotic tumor cell death. Since butyrate is a naturally occurring, inexpensive, and safe metabolite, its supplementation is a potentially feasible approach to overcome the resistance of ferroptosis-based therapies.

## Availability of data and materials

Raw ATAC-seq and RNA-seq data files are available in Genome Sequence Archive for Human database. ATAC-seq, accession number HRA002470, link: https://bigd.big.ac.cn/gsa-human/browse/HRA002470; RNA-seq, accession number HRA002471, link: https://bigd.big.ac.cn/gsa-human/browse/HRA002471.

## Funding

This work was supported by 10.13039/501100001809National Natural Science Foundation of China (82171730 to PX, 82204519 to YH), 10.13039/501100004731Natural Science Foundation of Zhejiang Province (LY20H160032 to PX, TGD23H160002 to YL), Medical Health Science and Technology Project of Zhejiang Province (2022KY363 to YH, 2020393247 to PX), Huzhou Science and Technology Foundation Project (2019GZ37 to YH).

## Ethics approval and consent to participate

All experiments involving human specimens were conducted under the approval from the Medical Ethics Committee of Sir Run Run Shaw Hospital of Zhejiang University (20220103–56), and the First Affiliated Hospital of Huzhou University (2021KYLL-Y-005). Informed consents were obtained from all participants.

## Author contributions

YH, PX conceived the study. YH, YL, ZZ, WS, XX, KW, KG, MS, HG, PX, ZZ performed the experiments and analyzed the data. YL, QS, XZ, KG, LH collected and processed clinical specimens. YH, RTM, PX, ZZ wrote and revised the manuscript. YH, PX, QC, WF provided financial or technical support. All authors read and approved the final manuscript.

## Declaration of competing interest

The authors declare no conflict of interest.

## Data Availability

Data will be made available on request.
