# Supplementary material for: Butyrate reverses ferroptosis resistance in colorectal cancer by inducing c-Fos-dependent xCT suppression
Source: Redox Biol. 2023 Jul 20;65:102822. doi: 10.1016/j.redox.2023.102822 (PMC10388208; doi:10.1016/j.redox.2023.102822)
Supplement: Multimedia component 1 [file mmc1.docx]

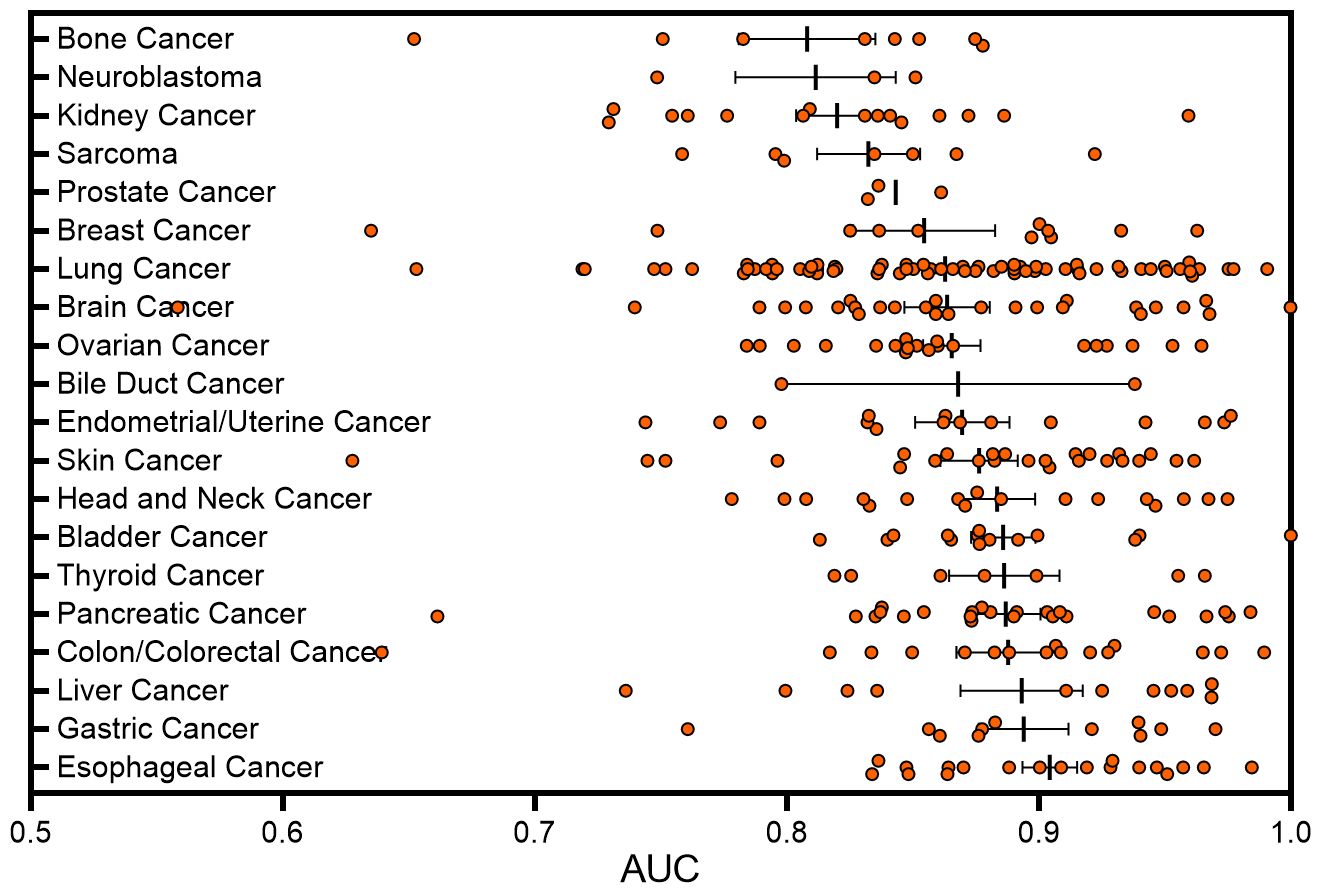


Supplementary Fig. S1. The sensitivity of 20 tumor types to erastin was analyzed using the DepMap database. AUC = Area Under Curve. Each dot represents a cell line from indicated cancer type. Cell lines with higher AUC values are more resistant to erastin.


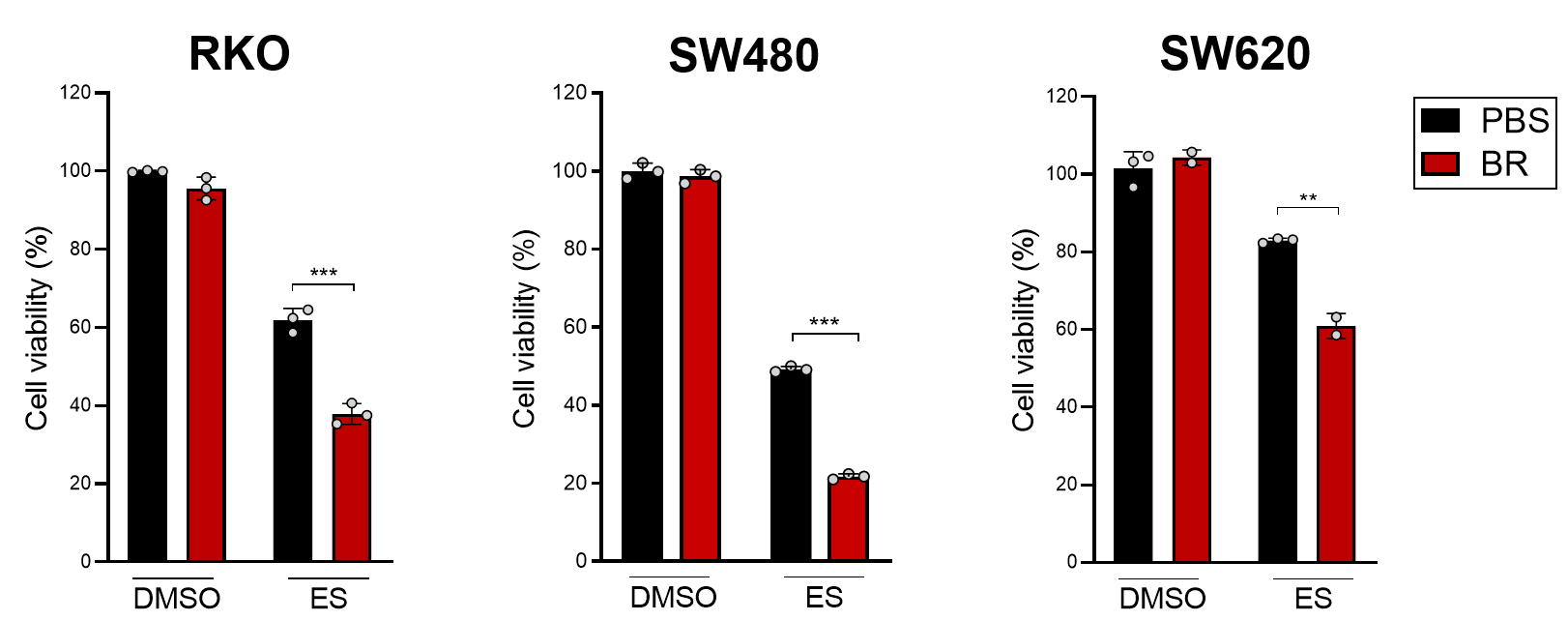


Supplementary Fig. S2. RKO, SW620 and SW480 cells were pretreated with butyrate (1 mM) followed by erastin (20 μM) treatment. Cell viability was evaluated by CCK8. Data are represented as the mean ± SD. ** *p* < 0.01; ****p* < 0.001, two-tailed unpaired Student’s *t* test.


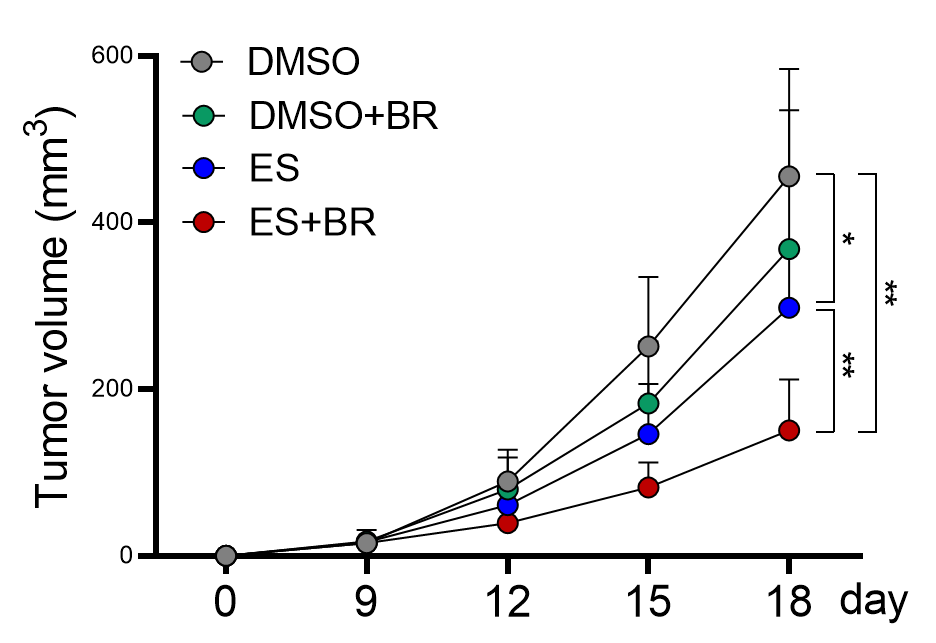


Supplementary Fig. S3. Butyrate sensitizes ferroptosis in a SW480 CRC model. SW480 tumor bearing mice were treated with erastin alone (30 mg/kg weight) or in combination with 100 mM butyrate in drinking water. Tumor growth was monitored. Data are represented as the mean ± SD. * *p* < 0.05; ***p* < 0.01, two-tailed unpaired Student’s *t* test.


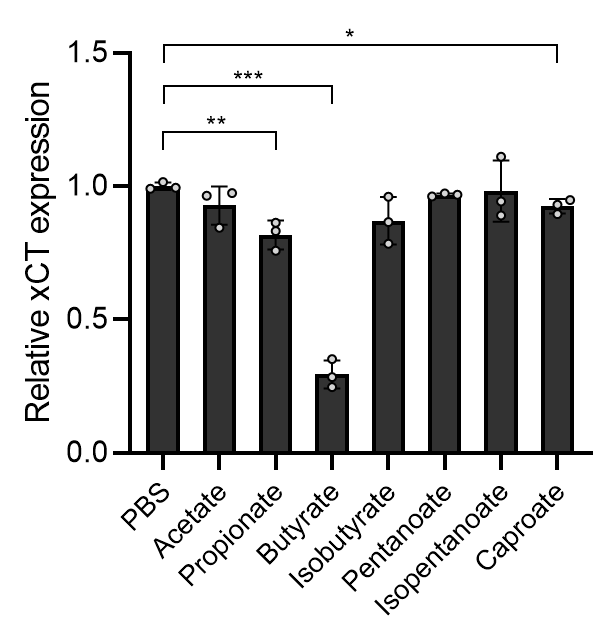


Supplementary Fig. S4. The effects of SCFAs on xCT expression. HCT116 cells were treated with 2 mM indicated SCFAs for 12 hours. The expression of xCT was evaluated by QPCR. Data are represented as the mean ± SD. ** *p* < 0.01; ****p* < 0.001, two-tailed unpaired Student’s *t* test.


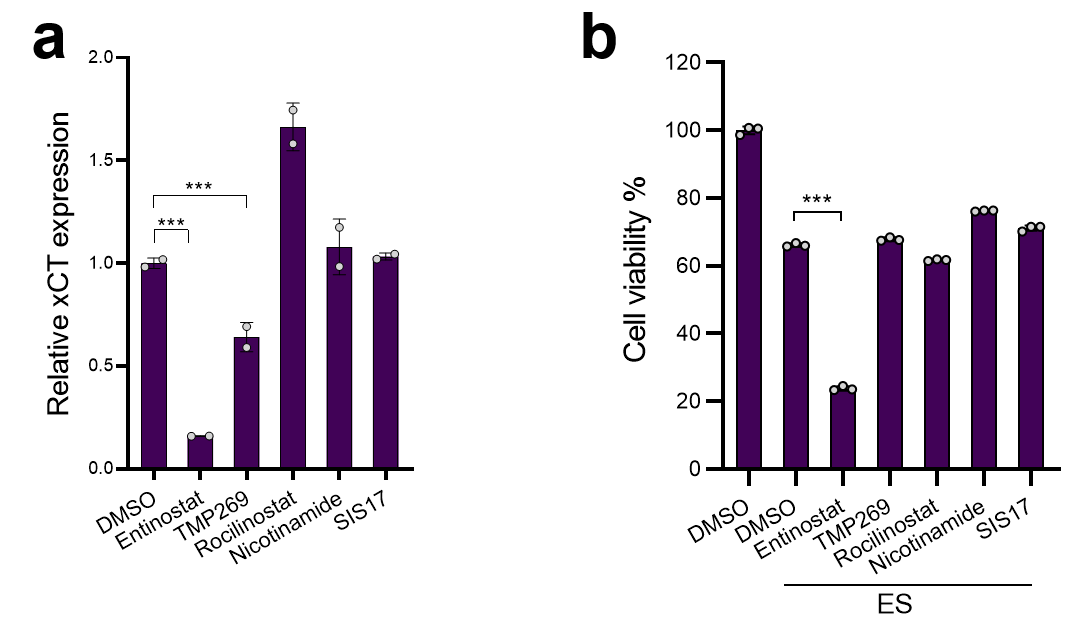


Supplementary Fig. S5. Butyrate increases c-Fos expression by inhibiting the activity of class I HDAC. **a,** HCT116 were treated with entinostat (class I HDAC inhibitor), TMP269 (class IIa HDAC inhibitor), rocilinostat (class IIb HDAC inhibitor), nicotinamide (class III HDAC inhibitor) or SIS17 (class IV HDAC inhibitor) for 12 hours (all at 10 μM). The expression of xCT was evaluated by QPCR. **b,** HCT116 cells were treated with erastin in the presence of indicated HDAC inhibitors for 24 hours. Cell viability was evaluated by CCK8. Data are represented as the mean ± SD. ^**^*p* < 0.01; *^***^p* < 0.001, two-tailed unpaired Student’s *t* test.

Supplementary Table 1. Primer sequences.

| **Gene** | **Sequences (5’-3’)** |
| --- | --- |
| human *ACTB* F | CATGTACGTTGCTATCCAGGC |
| human *ACTB* R | CTCCTTAATGTCACGCACGAT |
| human *SLC7A11* F | GCGTGGGCATGTCTCTGAC |
| human *SLC7A11* R | GCTGGTAATGGACCAAAGACTTC |
| human *FOS* F | CACTCCAAGCGGAGACAGAC |
| human *FOS* R | AGGTCATCAGGGATCTTGCAG |
| human *CD133* F | AGTCGGAAACTGGCAGATAGC |
| human *CD133* R | GGTAGTGTTGTACTGGGCCAAT |
| mouse *ACTB* F | GGCTGTATTCCCCTCCATCG |
| mouse *ACTB* R | CCAGTTGGTAACAATGCCATGT |
| mouse *SLC7A11* F | GGCACCGTCATCGGATCAG |
| mouse *SLC7A11* R | CTCCACAGGCAGACCAGAAAA |
| mouse *FOS* F | CGGGTTTCAACGCCGACTA |
| mouse *FOS* R | TTGGCACTAGAGACGGACAGA |
| mouse *LGR5* F | CCTACTCGAAGACTTACCCAGT |
| mouse *LGR5* R | GCATTGGGGTGAATGATAGCA |
